# Exposure assessment model of *Campylobacter* concentration in United States broiler processing plants

**DOI:** 10.1016/j.psj.2025.105758

**Published:** 2025-09-01

**Authors:** Rafael E. Rivera, Jinquan Wang, Abhinav Mishra, Harshavardhan Thippareddi, Sanjay Kumar, Manpreet Singh

**Affiliations:** a Department of Poultry Science, University of Georgia, Athens, GA 30602, USA; b Department of Poultry Science, Auburn University, 260 Lem Morrison Dr, Auburn, AL 36849, USA; c Department of Food Science & Technology, University of Georgia, Athens, GA 30602, USA; d Quality Technical Services and Food Safety, Niagara Bottling, Diamond Bar, CA 91765, USA

**Keywords:** *Campylobacter*, QMRA, Poultry parts, Comminuted poultry, Interventions

## Abstract

Poultry processing establishments play a critical role in mitigating *Campylobacter* contamination; a leading bacterial pathogen linked to poultry associated gastrointestinal illnesses in the United States. Quantitative microbial risk assessments (QMRA), suggest that reducing *Campylobacter* loads during processing can significantly mitigate public health risks. However, existing QMRAs often exclude contamination levels from cut-up parts and comminuted poultry products in their exposure assessments. To address this gap, a systematic review and meta-analysis were performed to estimate *Campylobacter* concentrations on whole birds, cut-up parts, and comminuted products, with and without interventions. Initial contamination levels derived from literature averaged 4.81 log_10_ CFU/mL. Log reductions (LR) across processing stages revealed significant decrease (*P* < 0.05) during scalding (LR: -2.86 log_10_ CFU/mL) and chilling (LR: -1.48 log_10_ CFU/mL). Baseline modeling of contamination levels showed concentrations of 1.38 log_10_ CFU/mL in whole birds, 0.79 log_10_ CFU/mL in cut-up parts, and 0.45 log_10_ CFU/mL in comminuted products. These estimates aligned with data from U.S. commercial establishments, where post-processing whole birds and parts exhibited 0.72 CFU/mL and ≤ 1 log_10_ CFU/mL, respectively. Chemical interventions improve *Campylobacter* reduction efficacy, particularly in cut-up parts and comminuted products. Single intervention strategies, such as post-chill immersion, achieved reductions up to 99.99%, while multi-hurdle approaches reduce pathogen levels to undetectable levels. These findings underscore the necessity of incorporating contamination data from poultry parts and comminuted products into QMRA frameworks to refine risk assessments and intervention strategies.

## Introduction

*Campylobacter,* a leading bacterial cause of gastrointestinal illness in the U.S., is strongly associated with poultry products consumption (Berghaus et al., 2013; Ellis-Iversen et al., 2012; Overesch et al., 2020). The Center for Disease Control and Prevention (CDC) estimates 1.5 million annual infections nationwide, with poultry-linked cases alone incurring $6.9 billion annual medical costs and loss of quality of life (CDC, 2022b; Scharff, 2020). Over 80 % of non-dairy foodborne *Campylobacter* illnesses are attributed to chicken, other seafood (such as shellfish) and turkey, predominantly chicken meat (IFSAC, 2021).

The U.S. Department of Agriculture, Food Safety and Inspection Service (USDA-FSIS) regulates poultry safety standards such as 1996 Pathogen Reduction; Hazard Analysis and Critical Control Points (PR/HACCP) rule, targeting contamination reduction at processing plants (USDA-FSIS, 1996). Baseline surveys under PR/HACCP revealed a decline in *Campylobacter* prevalence on chicken carcasses from 88.2 % (1995) to 18.3 % (2019) (Williams et al., 2021). However, recent USDA-FSIS data show persistent contamination: 20.94 % of carcasses, 16.75 % of cut-up parts, and 5.93 % of comminuted products tested positive in 2022 (USDA-FSIS, 2023). In addition, human illness rates have remained steady since PR/HACCP implementation, despite reduced prevalence (CDC, 2022).

Current regulatory strategies targeting *Campylobacter* have prioritized reducing pathogen prevalence at processing plants (USDA-FSIS, 2016, 2019). Emerging evidence, however, shows that lowering microbial load of foodborne pathogens rather than mere presence may more effectively mitigate public health risks (Sampedro et al., 2024). Quantitative microbial risk assessments (QMRA) are increasingly employed to analyze intervention strategies across food supply chain, enabling analysis of mitigation approaches (Dogan et al., 2019; Gonzalez et al., 2019). While there are several QMRA model that characterize *Campylobacter* contamination from farm-to-fork continuum, inconsistencies in model criteria and lack of integration across production stages hinder robust risk characterization (Chapman et al., 2016). Systematic reviews and meta-analysis (SR-MA) QMRAs can address these gaps by constructing baseline data to inform plant models and assess intervention efficacy.

Poultry processors predominantly rely on antimicrobial chemical interventions to meet performance standards (Wideman et al., 2016). SR-MAs provide critical insight into existing and novel interventions, guiding targeted implementation. However, current QMRAs and SR-MAs focus disproportionately on whole carcass data, neglecting cut-up parts and comminuted poultry (e.g., ground, chopped or shredded chicken) products representing the majority of U.S. poultry consumption (Chapman et al., 2016; Dogan et al., 2022, 2019; Golden and Mishra, 2020; Keener et al., 2004; Sahin et al., 2015). This oversight limits accurate exposure and dose-response assessments, as these products pose distinct contamination risks. Integrating *Campylobacter* population data from parts and comminuted chicken into QMRAs is essential to refine risk estimates and optimize interventions.

The objective of this study is to estimate the *Campylobacter* concentration levels in U.S. chicken parts and comminuted product reaching consumers through two approaches 1) Conduct an SR-MA to establish baseline *Campylobacter* levels in chicken parts and comminuted products from U.S. processing plants and 2) Simulate processing interventions to estimate their effectiveness in reducing pathogen concentrations in these products.

## Materials and methods

### Systematic review and meta-analysis

**Fig. 1:**
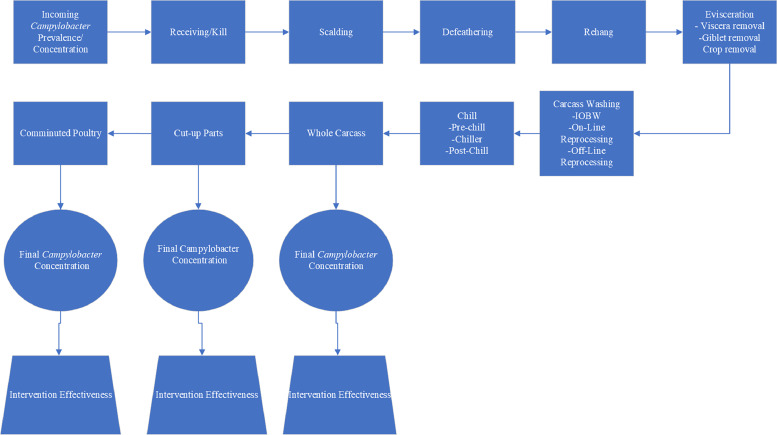
Flow diagram of poultry processing stages used for exposure assessment. *IOBW = inside/outside bird washer.

*Model Flow Chart.* A flow chart (Fig. 1) was developed to model *Campylobacter* population across poultry processing stages (receiving to grinding). Initial concentration of *Campylobacter* at the receiving stage, without intervention or chlorine treatment, was defined as baseline or control. The chicken processing stages include scalding, feather picking/ rehang, evisceration, carcass washing, immersion chilling, parts cut-up and grinding (comminuted) as standard operations of processing in the U.S. Final *Campylobacter* concentration data in cut-up parts, and comminuted products were analyzed to evaluate process and/or intervention efficacy. Additionally, the changes in *Campylobacter* population in each subsequent processing stage up to the grinding stage were estimated. Changes in bacterial load at each stage were quantified as log_10_ CFU/mL. Data for risk assessment inputs were obtained through a systematic review of literature and meta-analysis.

*Literature Search and Inclusion Criteria.* A systematic review was adapted from Golden and Mishra (2020), and Sargeant and O'Connor (2014) to address the following research question:

1. How does Campylobacter contamination on broiler carcasses change at each stage of processing from receiving to chicken parts and comminuted product in the U.S.?
2. What is the efficacy of chemical intervention and processing equipment on reducing Campylobacter contamination and their interactions in the U.S.?

To address this question, the Web of Science (www.webofknowledge.com) and PubMed (https://pubmed.ncbi.nlm.nih.gov/) were searched using keywords aimed at addressing the research questions: (“*Campylobacter*” or “*Campylobacter jejuni*” or “*C. jejuni*”), AND (“United States” or “U.S.”) AND (“Poultry” or “Broiler” or “Chicken”) and “Intervention” and “Processing” and (“Concentration” or “Isolation”) and data up to January 2023 was retrieved. In the absence of geographic description of the study, the location was inferred by the first and corresponding address. Additional studies were identified by searching review articles or other reference lists by hand. Inclusion criteria for accepting data includes, 1) Peer- reviewed, English-language primary research studies, excluding reviews; 2) Conducted in U.S. commercial/pilot plants; 3) Reported *Campylobacter* concentrations (log_10_ CFU/mL) in whole carcasses, parts, or comminuted products; 4) Tested interventions (chemical/equipment-based) with before-after or challenge study designs. All references were managed by the EndNote citation manager (Endnote 20, Clarivate Analytics, Philadelphia, PA). Duplicates were removed from EndNote by using the “find duplicates” function or manually.

Challenge studies were included only where commercial data was insufficient, with caveats regarding potential overestimation of intervention efficacy. Non-U.S. studies, review, and non-English articles were excluded.

*Data Extraction and Conversion.* Extracted data included processing stage, intervention type, application method, sample size, mean concentration, and variability metrics (standard deviation, standard error, confidence intervals). Missing standard deviation values were calculated from reported standard error/ confidence intervals using standard formulas (Higgins, 2008). The data were directly collected if the table is available, whereas the Plot Digitizer tool (Plot Digitizer, 3.1.5, 2024, https://plotdigitizer.com) was used to extract the mean and error values from the figures. All *Campylobacter* concentrations are reported as log_10_ CFU/mL. Conversions to log_10_ CFU/mL from log_10_ CFU/g, log_10_ CFU/cm^2^, or log_10_ CFU/carcass were performed using the conversions recommended by Appendix A of the Joint Food and Agriculture Organization/ World Health Organization Risk Management Tool for the Control of *Campylobacter* and *Salmonella* in Chicken Meat (JEMRA, 2009).

The concentration changes across processing stages and interventions were quantified using Log Change (LC), calculated as $LC=C-C_{i}$ where C and $C_{i}$ represent post and pre-processing concentrations, respectively. A LC < 0 indicates a decrease in concentration, a LC = 0 indicates no change, and a LC > 0 indicates an increase in concentration. This metric characterizes contamination dynamics during processing (Dogan et al., 2022).

*Quality Assessment of Included Studies.* While systematic reviews and meta-analysis typically assess study quality, quality scoring was omitted here to avoid selection bias stemming from variability in scoring methodologies and their influence on meta-analytical interpretations (Stone et al., 2019).

*Data Analysis.* A random effects meta-analysis was conducted to determine *Campylobacter* concentrations, LC values, and intervention efficacy across poultry processing stages. Analyses adhered to Preferred Reporting Items for Systematic Review and Meta-Analyses (Page et al., 2021) guidelines, with results visualized as tables and forest plots. The meta-analysis was conducted using the meta package (Schwarzer, 2007) in R software 4.0.2 (R Core Team, 2021). Summary statistics included 95 % confidence intervals (CI), between-study variance (τ^2^), and heterogeneity (I^2^) interpreted as follows: The value of I^2^ up to 40 % was considered low, 30–60 % was considered moderate, 50–90 % was considered substantial, and beyond 75 % was considered high (Deek et al., 2019).

*Publication Bias Assessment.* Funnel plot asymmetry test for publication bias requires ≥10 studies and low heterogeneity (I^2^ < 50 %). As these criteria was not met, publication bias was not performed.

### Exposure assessment

*Processing Plant Module Overview*. The first objective of this module was to estimate *Campylobacter* concentration levels (log_10_ CFU/ mL) in whole birds, cut-up parts, and comminuted poultry under chlorine or no-interventions conditions, spanning stages from receiving to griding. The second objective was to estimate interventions efficacy in reducing *Campylobacter* during processing.

*Baseline Concentration Estimate.* Initial *Campylobacter* concentration at the receiving stage were obtained from SR-MA results. The processing model incorporated standard U.S. processing stages (scalding, feather picking/ rehang, evisceration, carcass washing, carcass chilling, parts cut up and grinding), with baseline inputs pooled from trials lacking reported interventions or reported chlorine use. Chlorine was used as part of the baseline since this intervention has historically been used for pathogenic bacterial control. It was assumed that commercial processing plant studies without reported use of interventions in control trials were using chlorine at the time of sampling.

LC values, modeled as normal distribution, were simulated using Monte Carlo simulation by Latin Hypercube Sampling with 10000 iterations using @Risk (version 8.4.1 (Build10), Palisade Company LLC, New York, USA). Baseline outputs for whole birds, chicken cut-up parts, and comminuted chicken were obtained for intervention efficacy analysis.

*Baseline Validation.* Pre- and post-processing *Campylobacter* concentrations recovered from routine testing of 31 U.S. commercial processing over the period from 2018 to 2024 were analyzed. Samples with a limit of detection (LOD) of 1 CFU/mL included pre-scalder, post-chill and cut-up parts. Concentration variations across parts (breast fillets, thighs and wings, etc.) were analyzed using one-way ANOVA and were further separated using Tukey HSD (*P* < 0.05) method in R (R Core Team, 2021).

*Intervention Efficacy Analysis.* Single interventions (such as replacing immersion chilling with air chilling) or added steps (e.g., post-chill dips) obtained from the SR-MA were evaluated for its efficacy. Results from the interventions were expressed as 1. Mean *Campylobacter* concentration in CFU/mL with its 95 % CI and 2. Intervention efficacy calculated using equation:

$$Intervention\;efficacy=\;\frac{Concentration_{baseline}-\;Concentration_{intervention}}{Concentration_{baseline}}\;X\;100$$

where $Concentration_{baseline}$ refers to the *Campylobacter* concentration for either whole birds, cut-up parts or comminuted chicken and $Concentration_{intervention}$ refers to the *Campylobacter* concentration for the alternative intervention scenario. Multiple intervention scenarios (pre- and post-chill) were also evaluated for cumulative effects on *Campylobacter* concentrations in whole birds, cut-up parts and comminuted chicken.

## Results

### Systematic review and meta-analysis

**Fig. 2:**
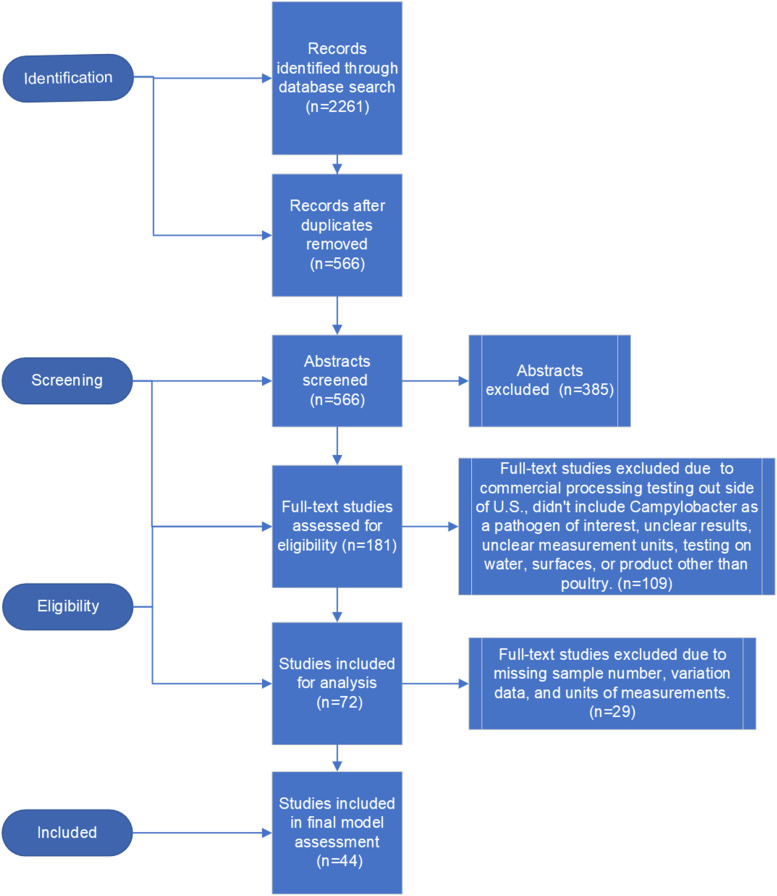
Flow chart of the systematic review process.

*Search Results.* The initial search criteria produced 2,261 studies. After removing duplicates and screening the titles and abstracts, 181 records were retained for full text screening. After full text screening, 72 records were retained for analysis. 29 records were excluded due to missing sample number, variation data, and units of measurements that could not be converted to the log_10_ CFU/mL. A total of 18 commercial plants before/after studies, 10 pilot plant before/after studies, 5 pilot plant challenge studies, and 11 lab scale challenge studies, totaling 44 studies were included for the risk assessment. The overview of the systematic review process is illustrated in Fig. 2.

*Characteristics of Included Studies.* The characteristics of the included studies in the meta-analysis and risk assessment model are presented in Table 1. 6 studies reporting *Campylobacter* concentrations at receiving or prior to the scalding step were used to determine an incoming load. 27 studies used chlorine or did not report an intervention in its control group. The control group consisted of trials without reported use of an intervention or chlorine use. Chlorine was used as part of the baseline since this intervention has historically been used for pathogenic bacterial control. It was assumed that commercial processing plant studies without reported use of interventions in control trials were using chlorine at the time of sampling. These were used to determine the baseline *Campylobacter* concentration and concentration change for each processing stage.

**Table 1:** Characteristics of Included Studies from Systematic Review.

Reference	Processing Stage	Study type	Location	Equipment	Treatment	Sample Type	Unit of Enumeration
Berghaus et al. (2013)	Receiving (Incoming Load)	Commercial plant before/after study	Receiving	Transport coops	None	Carcass Rinse	log MPN/carcass
Berrang, Buhr, et al. (2000)	Receiving (Incoming Load)	Commercial plant before/after study	Kill step	Blood tunnel	None	Carcass and Viscera Samples	log CFU/g
Kotula and Pandya (1995)	Receiving (Incoming Load)	Commercial plant before/after study	Scalding	Scalder	None	Skin/Feathers/Feet Samples	log CFU/g
Mead et al. (1995)	Receiving (Incoming Load)	Commercial plant before/after study	Kill step	Automatic killing machine	None	Skin/ Cecal Samples	log CFU/g
Potturi-Venkata et al. (2007)	Receiving (Incoming Load)	Commercial plant before/after study	Farm samples	None	None	Fecal Samples	log CFU/mL
Stern and Robach (2003)	Receiving (Incoming Load)	Commercial plant before/after study	Farm samples	None	None	Fecal Samples	log CFU/g
DeVillena et al. (2022)	Receiving (Incoming Load)	Commercial plant before/after study	Receiving	None	None	Carcass Rinse	log CFU/mL
Berrang et al. (2003)	Scalding	Commercial plant before/after study	Scalding	Scalding bath	None	Carcass Rinse	log CFU/mL
Berrang, Windham, et al. (2011)	Scalding	Commercial plant before/after study	Scalding	Scalding bath	None/ High pH	Carcass Rinse	log CFU/mL
Berrang and Dickens (2000)	Scalding	Commercial plant before/after study	Scalding	Spray Cabinet	Chlorine	Carcass Rinse	log CFU/mL
Berrang et al. (2001)	Feather Picking	Pilot plant before/after study	Post-Pick	Feather Picker	None/Chlorine/Cloacal Plug	Sponge	log CFU/mL
Berrang and Dickens (2000)	Feather Picking	Commercial plant before/after study	Post-Pick	Feather Picker Spray	Chlorine	Carcass Rinse	log CFU/mL
Berrang, Dickens, et al. (2000)	Feather Picking	Pilot plant before/after study	Post-Pick	Scalding Tanks and Spray	Post Immersion rescald/ Post spray rescald	Carcass Rinse	log CFU/mL
Berrang et al. (2006a)	Feather Picking	Pilot plant before/after study	Post-Pick	Feather Picker	Cloacal Wash with Vinegar	Sponge	log CFU/mL
Berrang et al. (2006b)	Feather Picking	Pilot plant before/after study	Post-Pick	Feather Picker	Cloacal Wash with Organic Acids	Sponge	log CFU/mL
Berrang, Meinersmann, et al. (2011)	Feather Picking	Commercial plant before/after study	Post pick	Feather Picker Spray	None/ ClO_2_	Carcass Rinse	log CFU/mL
Berrang, Windham, et al. (2011)	Feather Picking	Commercial plant before/after study	Post-Pick	Post pick dip tank	Chlorine	Carcass Rinse	log CFU/mL
Berrang et al. (2018)	Feather Picking	Pilot plant before/after study	Post-Pick	Feather Picker	None/Cloacal Plug	Sponge	log CFU/mL
Musgrove et al. (1997)	Feather Picking	Commercial plant before/after study	Post pick	Scald and pickers	Cloacal plug	Carcass Rinse	log CFU/mL
Berghaus et al. (2013)	Rehang	Commercial plant before/after study	Rehang	Rehanger	None	Carcass Rinse	log MPN/ Carcass
Berrang et al. (2007)	Rehang	Commercial plant before/after study	Rehang	Rehanger	Chlorine	Carcass Rinse	log CFU/mL
DeVillena et al. (2022)	Rehang	Commercial plant before/after study	Rehang	Rehanger	Chlorine/ PAA	Carcass Rinse	log CFU/mL
Northcutt et al. (2003)	Evisceration	Pilot plant before/after study	Post-Evisceration	In-line evis equipment	None	Carcass Rinse	log CFU/mL
Meredith et al. (2013)	Evisceration	Commercial plant before/after study	Post-Evisceration	In-line evis equipment	None/Cloacal Wash with Organic Acids	Carcass Swabs	log CFU/cm^2^
Berrang and Dickens (2000)	Evisceration	Commercial plant before/after study	Post-Evisceration	In-line evis equipment	Chlorine	Carcass Rinse	log CFU/mL
DeVillena et al. (2022)	Evisceration	Commercial plant before/after study	Post-Evisceration	In-line evis equipment	Chlorine	Carcass Rinse	log CFU/mL
Oyarzabal et al. (2004)	Carcass Wash	Commercial plant before/after study	Carcass washing	IOBW	None	Carcass Rinse	log CFU/ml
Berrang and Dickens (2000)	Carcass Wash	Commercial plant before/after study	Carcass washing	IOBW	Chlorine	Carcass Rinse	log CFU/ml
Berghaus et al. (2013)	Carcass Wash	Commercial plant before/after study	Carcass washing	IOBW	None	Carcass Rinse	log MPN/carcass
James et al. (2007)	Carcass Wash	Pilot plant challenge study	Carcass washing	Pre-Chill Spray	Steam	Skin Rinse	log CFU/cm^2^
Li et al. (2002)	Carcass Wash	Pilot plant challenge study	Carcass washing	IOBW	Chlorine/High Temperature Wash	Carcass Rinse	log MPN/carcass
Zhang et al. (2011)	Carcass Wash	Commercial plant before/after study	Carcass washing	Pre-Chill Spray	CPC	Carcass Rinse	log CFU/mL
DeVillena et al. (2022)	Carcass Wash	Commercial plant before/after study	Carcass washing	IOBW/Pre-Chill Spray	Chlorine/PAA	Carcass Rinse	log CFU/mL
Zhang et al. (2011)	Carcass Chill	Commercial plant before/after study	Chiller	Immersion chiller	Chlorine/Air Chill	Carcass Rinse	log CFU/mL
Stern and Robach (2003)	Carcass Chill	Commercial plant before/after study	Chiller	Immersion chiller	Chlorine	Carcass Rinse	log CFU/ carcass
Potturi-Venkata et al. (2007)	Carcass Chill	Commercial plant before/after study	Chiller	Immersion chiller	None	Carcass Rinse	log CFU/mL
Oyarzabal et al. (2004)	Carcass Chill	Commercial plant before/after study	Chiller/ Post-Chill	Immersion chiller/ Post-Chill Tank	ASC	Carcass Rinse	log CFU/mL
Northcutt et al. (2006)	Carcass Chill	Pilot plant before/after study	Chiller	Immersion chiller	None	Carcass Rinse	log CFU/mL
Northcutt, Smith, et al. (2008)	Carcass Chill	Commercial plant before/after study	Chiller	Immersion chiller	Chlorine	Carcass Rinse	log CFU/mL
Northcutt, Cason, et al. (2008)	Carcass Chill	Pilot plant before/after study	Chiller	Immersion chiller	None	Carcass Rinse	log CFU/mL
Huezo et al. (2007)	Carcass Chill	Pilot plant before/after study	Chiller	Immersion chiller/ Air Chiller	None	Carcass Rinse	log CFU/mL
Cason et al. (1997)	Carcass Chill	Commercial plant before/after study	Chiller	Immersion chiller	None	Carcass Rinse	log CFU/ carcass
Berrang and Dickens (2000)	Carcass Chill	Commercial plant before/after study	Chiller	Immersion chiller	Chlorine	Carcass Rinse	log CFU/mL
Berrang et al. (2008)	Carcass Chill	Pilot plant before/after study	Chiller	Immersion chiller/ Air Chiller	None	Carcass Rinse	log CFU/mL
Berrang et al. (2007)	Carcass Chill	Commercial plant before/after study	Chiller/ Post-Chill	Immersion chiller	Chlorine	Carcass Rinse	log CFU/mL
Berghaus et al. (2013)	Carcass Chill	Commercial plant before/after study	Pre-Chill/ Chiller	IOBW/ Immersion Chiller	None	Carcass Rinse	log MPN/carcass
Smith et al. (2015)	Carcass Chill	Lab challenge study	Post-Chill	Dip Tank/Spray Cabinet	None/ Chlorine/ PAA	Carcass Rinse	log CFU/mL
Nagel et al. (2013)	Carcass Chill	Pilot plant challenge study	Post-Chill	Dip Tank	None/Chlorine/PAA/ Lysozyme	Carcass Rinse	log CFU/mL
DeVillena et al. (2022)	Carcass Chill	Commercial plant before/after study	Post-Chill	Dip Tank	Chlorine/ PAA	Carcass Rinse	log CFU/mL
Bourassa et al. (2021)	Parts	Lab challenge study	Post-Chill processing	Dip Tank/Spray Cabinet	None/HP/ PAA	Wing Rinse	log CFU/mL
Gonzalez et al. (2021)	Parts	Lab challenge study	NA	Dip Tank/Spray Cabinet	None/SSS/FA/ PAA	Wing Rinse	log CFU/mL
Gunther et al. (2016)	Parts	Lab challenge study	NA	UV	UV	Skin Homogenate	log CFU/mL
Haughton et al. (2012)	Parts	Lab challenge study	NA	UV/PEF	UV/PEF	Skin/Boneless Breast	log CFU/g
Hinton and Ingram (2005)	Parts	Lab challenge study	NA	Spray Cabinet	TPP	Skin Rinse	log CFU/mL
Kataria et al. (2020)	Parts	Lab challenge study	NA	Dip Tank	PAA	Wing Rinse	log CFU/g
Kumar et al. (2020)	Parts	Lab challenge study	NA	Dip Tank/Spray Cabinet	PAA	Boneless Breast Rinse	log CFU/mL
Sarjit and Dykes (2015)	Parts	Lab challenge study	NA	Spin Chiller	None/Chlorine/ TSP	Breast Meat with Skin	log CFU/ cm2
Vaddu et al. (2021)	Parts	Lab challenge study	NA	Dip Tank	PAA	Wing Rinse	log CFU/mL
Zang et al. (2018)	Parts	Pilot plant challenge study	NA	Dip Tank	None/Chlorine/ ASC/ PAA/ CPC	Various Parts	log CFU/mL
DeVillena et al. (2022)	Parts	Commercial plant before/after study	Post-Cut-Up	Dip Tank	Chlorine/PAA	Wing Rinse	log CFU/mL
Chen et al. (2014)	Comminuted	Pilot plant challenge study	Cut up /Grinding	Dip Tank/ Spray	None/Chlorine/ PAA/ CPC	Ground Chicken Breast/Thighs	log CFU/g
Park et al. (2017)	Comminuted	Lab challenge study	NA	Dip tanks	None/Chlorine/ PAA	Ground Chicken Breast	log CFU/g

27 studies reported intervention trials other than the control group to control *Campylobacter* concentration. The studies reported the use of 18 different treatment types against *Campylobacter* concentration. The interventions that were included for meta-analysis were acidified sodium chlorite (ASC *n* = 3), air chill (AC *n* = 1), cloacal plug (CP *n* = 3), cloacal wash (CW *n* = 3), cetylpiridium chloride (CPC *n* = 3), chlorine dioxide (ClO_2_ *n* = 1), high scalding pH (*n* = 1), peroxyacetic acid (PAA *n* = 10), lysozyme (*n* = 1), trisodium phosphate (TSP *n* = 1), hydrogen peroxide (*n* = 1), formic acid (FA *n* = 1), sulfuric acid + sodium sulfate solution (SSS *n* = 1), rescald (*n* = 1), steam (*n* = 1), tripotassium phosphate (TPP *n* = 1), ultraviolet light (UV *n* = 2), pulsed electric field (PEF *n* = 1). Chemical applications were applied either through an immersion application (dip tank) (*n* = 25) or a spray application (*n* = 17).

**Fig. 3:**
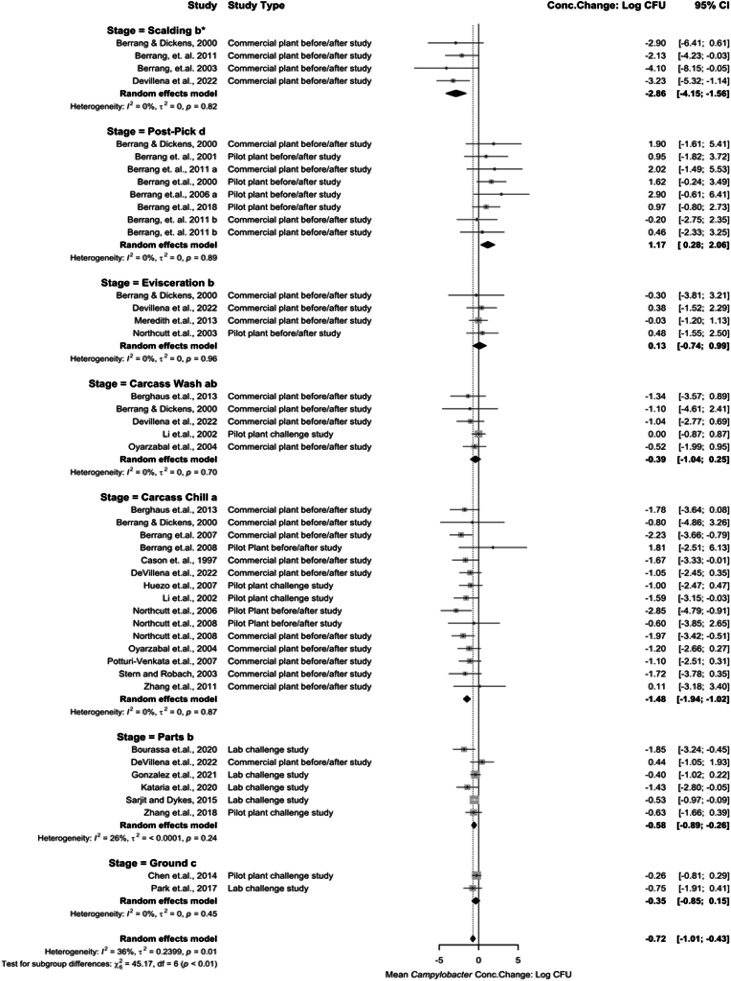
Campylobacter concentration change per stage without reported interventions or chlorine. The random effects model results represent the mean concentration change per stage for the control group that includes studies without reported interventions or chlorine. Results > 0 indicate an increase in concentration. Results < 0 indicate a decrease. Results = 0 indicate no change. Distinct letters next to the stage description indicate statistically significant differences (*P* < 0.05).

*Meta-analysis for Campylobacter Concentration Changes per Processing Stage for Control Group.* Receiving (incoming load) was estimated at 4.81 log_10_ CFU/mL (95 % CI: 3.91 to 5.72). *Campylobacter* concentration change was obtained for 7 stages (Fig. 3). Scalding being −2.86 log_10_ CFU/mL (95 % CI: −4.15 to −1.56), Feather Pick being 1.17 log_10_ CFU/mL (95 % CI: 0.28 to 2.06), Evisceration being 0.13 log_10_ CFU/mL (95 %CI: −0.74 to 0.99), Carcass Wash (IOBW) being −0.39 log_10_ CFU/mL (95 % CI: −1.04 to 0.25), Carcass Chill (Immersion Chiller) being −1.48 log_10_ CFU/mL (95 %CI: −1.94 to −1.02), Cut-Up Parts being −0.58 log_10_ CFU/mL (95 % CI: −0.89 to −0.26), and Comminuted (Ground) being −0.35 log_10_ CFU/mL (95 % CI: −0.85 to 0.15).

Scalding represents the highest concentration reduction. Post-Pick represents a significant increase (*P* < 0.05) in *Campylobacter* concentrations. Significant reductions are represented in immersion chilling (*P* < 0.05). Subsequent processes for cut up or grinding also represent reductions in *Campylobacter* concentrations. Low heterogeneity existed in the analysis between groups (I^2^ = 0 %, *P* > 0.05).

**Fig. 4:**
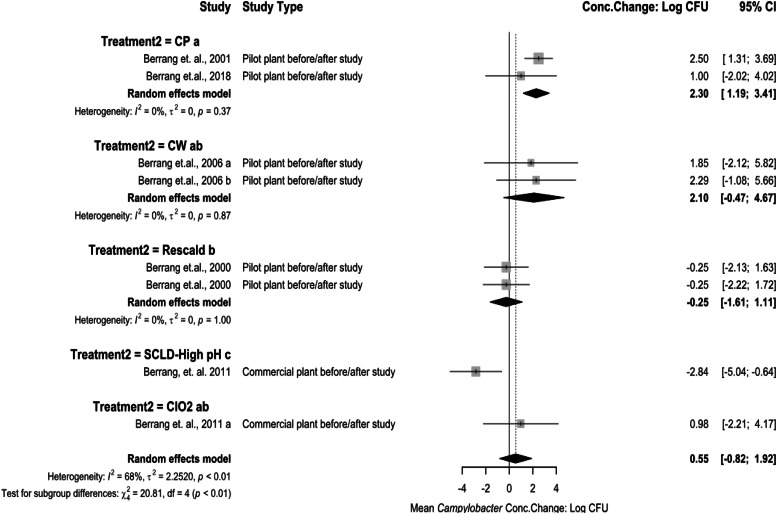
Campylobacter Concentration Change of Interventions at Scalding and Feather Picking. The random effects model results represent the mean concentration change of included interventions at the scald and feather picking stages. Results > 0 indicate an increase in concentration. Results < 0 indicate a decrease. Results = 0 indicate no change. Distinct letters next to the stage description indicate statistically significant differences (*P* < 0.05). Abbreviations: CP = cloacal plug, CW = cloacal wash, SCLD-High pH = high pH scald, ClO2 = chlorine dioxide.

**Fig. 5:**
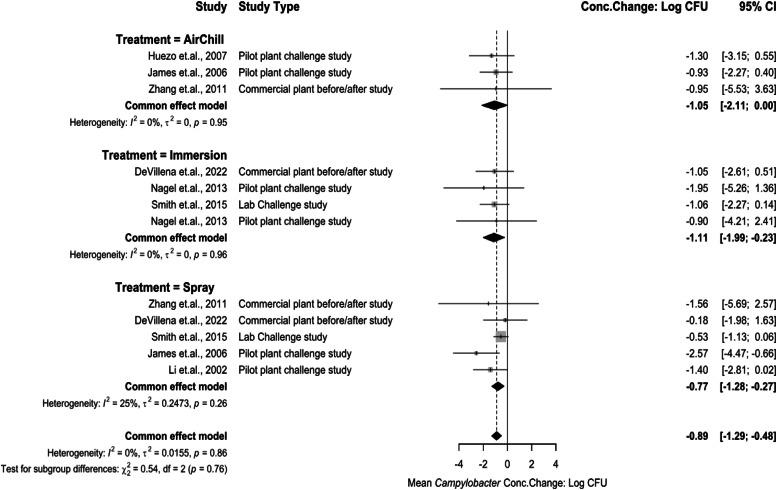
Campylobacter concentration change of intervention application at pre-chill and air chilling stages. The random effects model results represent the mean concentration change of included interventions at the pre-chill and air chill picking stages. Results > 0 indicate an increase in concentration. Results < 0 indicate a decrease. Results = 0 indicate no change. Distinct letters next to the treatment description indicate statistically significant differences (*P* < 0.05).

*Meta-analysis for Interventions Against Campylobacter.* Several pre-chill and post-chill interventions were compared for their *Campylobacter* concentration change. Pre-chill interventions against *Campylobacter* included analysis for scalding and feather picking applications (Fig. 4) and pre-chill chemical applications (Fig. 5). A treatment to increase pH was used at the scalding stage. The concentration change being −2.84 log_10_ CFU/mL (95 % CI: −5.04 to −0.64). A ClO_2_ wash used at the feather picking stage, along with CW, and CP were the main interventions applied prior to the rehang stage. The concentration changes for ClO_2_ being 0.98 log_10_ CFU/mL (95 % CI: −2.21 to 4.17), CW being 2.09 log_10_ CFU/mL (95 % CI: −0.47 to 4.67), and CP being 2.30 log_10_ CFU/mL (95 % CI: 1.19 to 3.41). A rescald application was also included after feather pick with concentration change being −0.25 log_10_ CFU/mL (95 % CI: −1.61 to 1.11). The test for subgroup differences resulted in the high pH treatment being significantly different in its reduction capability compared to rescald (*P* < 0.05). The test for subgroup differences among the feather picking interventions resulted in ClO_2_ treatment being significantly different (*P* < 0.05) compared to CP and CW. Low heterogeneity existed in the analysis between groups (I^2^ = 0 %, *P* > 0.05).

Immersion and spray applications were evaluated prior to the chilling stage. Immersion application concentration change being −1.11 log_10_ CFU/mL (95 % CI: −1.99 to −0.23), spray applications being −0.77 log_10_ CFU/mL (95 % CI: −1.28 to −0.27). The effect of air chilling on *Campylobacter* concentration change was evaluated with the effect being −1.05 log_10_ CFU/mL (95 %CI: −2.11 to 0.00). The test for subgroup differences was not significant (*P* > 0.05), and there was low heterogeneity between studies, but not statistically significant (I^2^ = 0 %, *P* > 0.05).

**Fig. 6:**
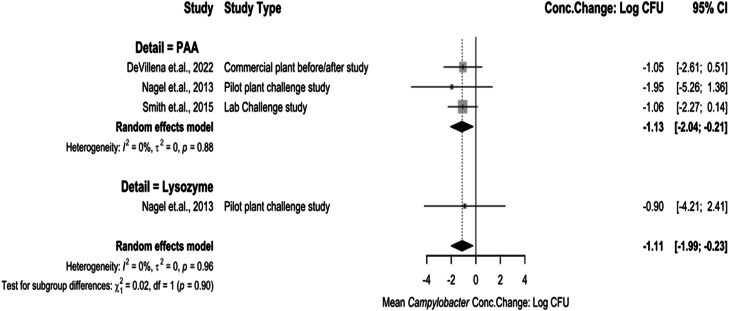
Campylobacter concentration change of immersion interventions at pre-chill stage. The random effects model results represent the mean concentration change of included immersion interventions at the pre-chill stage. Results > 0 indicate an increase in concentration. Results < 0 indicate a decrease. Results = 0 indicate no change. Distinct letters next to the treatment. description indicate statistically significant differences (*P* < 0.05). Abbreviations: PAA = peroxyacetic acid.

The chemical interventions analyzed as immersion interventions at pre-chill were PAA and Lysozyme (Fig. 6). PAA as an immersion treatment concentration change being −1.13 log_10_ CFU/mL (95 % CI: −2.04 to −0.21). Lysozyme concentration change being −0.90 log_10_ CFU/mL (95 % CI: −4.21 to 2.41). The test for subgroup differences was not significant (*P* > 0.05), and there was low heterogeneity between studies, but not statistical significance (I^2^ = 0 %, *P* > 0.05).

**Fig. 7:**
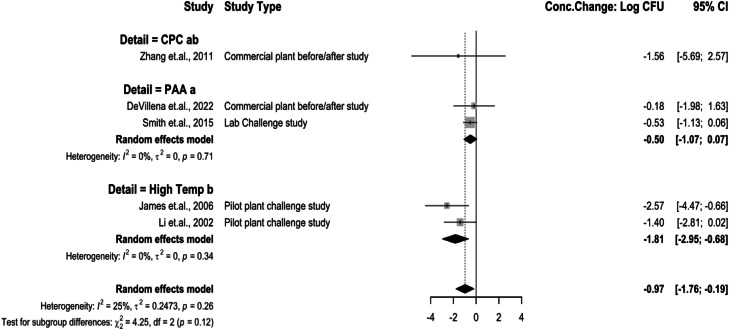
Campylobacter concentration change for spray interventions at pre-chill stage. The random effects model results represent the mean concentration change of included spray interventions at the pre-chill stage. Results > 0 indicate an increase in concentration. Results < 0 indicate a decrease. Results = 0 indicate no change. Distinct letters next to the treatment description indicate statistically significant differences (*P* < 0.05). *Abbreviations: CPC = cetylpiridium chloride, PAA = peroxyacetic acid, High Temp = high temperature wash or steam.*

The chemical interventions analyzed as spray interventions at pre-chill were PAA, CPC and treatments with high temperature water and steam (Fig. 7). PAA as a spray treatment concentration change being −0.50 log_10_ CFU/mL (95 % CI: −1.07 to 0.07). CPC concentration change being −1.56 log_10_ CFU/mL (95 % CI: −5.69 to 2.57). High temperature and steam spray treatment concentration changes being −1.81 log_10_ CFU/mL (95 % CI: −2.95 to −0.68). The test for subgroup differences was significant (*P* < 0.05), and there was low heterogeneity between studies, but not statistically significant (I^2^ = 0 %, *P* > 0.05).

**Fig. 8:**
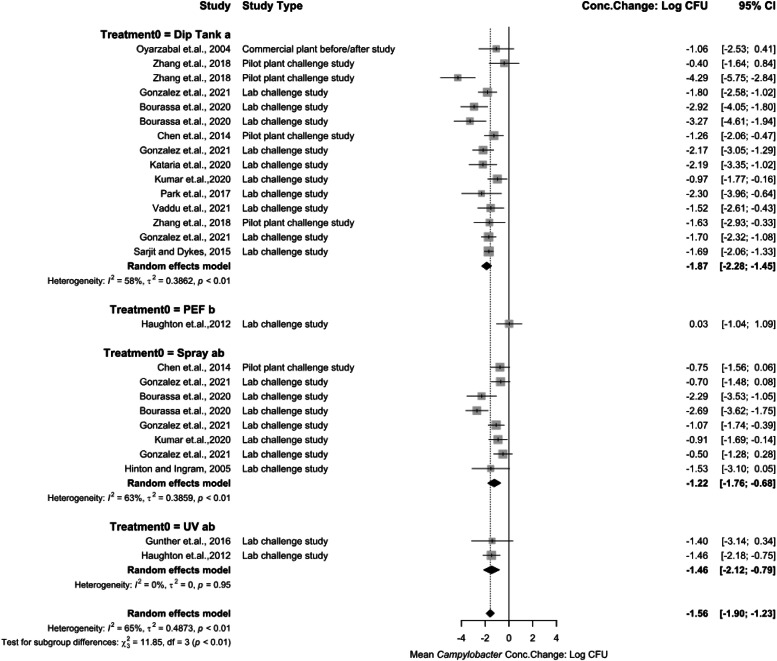
Campylobacter concentration change of intervention applications at post-chill stage. The random effects model results represent the mean concentration change of included spray interventions at the pre-chill stage. Results > 0 indicate an increase in concentration. Results < 0 indicate a decrease. Results = 0 indicate no change. Distinct letters next to the stage description indicate statistically significant differences (*P* < 0.05). Abbreviations: PEF = pulsed electric field, UV= ultraviolet light.

Several post-chill interventions were categorized as immersion or spray applications (Fig. 8). Interventions, such as UV and PEF, were also extracted from systematic review and included as additional physical interventions for further study. Post-chill immersion application concentration change being −1.87 log_10_ CFU/mL (95 % CI: −2.28 to −1.45), spray applications being −1.22 log_10_ CFU/mL (95 % CI: −1.76 to −0.69). PEF concentration change being 0.03 log_10_ CFU/mL (95 % CI: −1.04 to 1.09) and UV being −1.46 log_10_ CFU/mL (95 % CI: −2.12 to −0.79). Immersion treatments have a higher concentration change effect at reducing *Campylobacter* concentrations in chicken products as a post chill application. The overall reduction is not statistically significant (*P* > 0.05) from spray and UV applications. PEF was less effective as a post-chill intervention being statistically significant (*P* < 0.05) from immersion, spray, and UV. Heterogeneity was high between immersion (I^2^ = 58 %, *P* < 0.05) and spray groups (I^2^ = 63 %, *P* < 0.05) indicating that the intervention application methods as overall groups should be interpreted carefully.

**Fig. 9:**
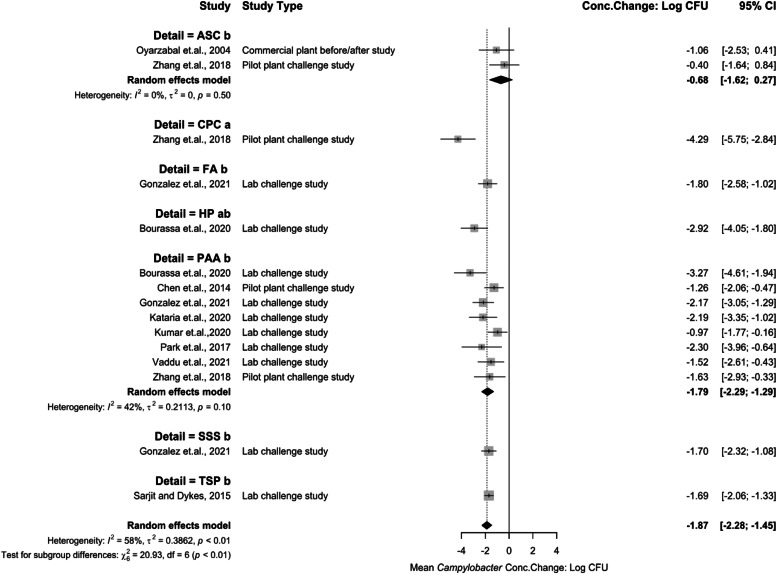
Campylobacter concentration change for immersion interventions at post-chill stage. The random effects model results represent the mean concentration change of included immersion interventions at the post-chill stage. Results > 0 indicate an increase in concentration. Results < 0 indicate a decrease. Results = 0 indicate no change. Distinct letters next to the treatment description indicate statistically significant differences (*P* < 0.05). Abbreviations: ASC = acidified sodium chlorite, CPC = cetylpiridium chloride, FA = formic Acid, HP = hydrogen peroxide, PAA = peroxyacetic acid, SSS= sulfuric acid + sodium sulfate solution, TSP = trisodium phosphate.

There were several chemical interventions analyzed for mean *Campylobacter* concentration change as post-chill immersion treatments (Fig. 9). ASC being −0.68 log_10_ CFU/mL (95 % CI: −1.62 to 0.27), CPC being −4.29 log_10_ CFU/mL (95 % CI: −5.75 to −2.84), FA being −1.80 log_10_ CFU/mL (95 % CI: −2.58 to −1.02), HP being −2.92 log_10_ CFU/mL (95 % CI: −4.05 to −1.80),PAA being −1.79 log_10_ CFU/mL (95 % CI: −2.29 to −1.29), SSS being −1.70 log_10_ CFU/mL (95 % CI: −2.32 to −1.08), and TSP being −1.69 log_10_ CFU/mL (95 % CI: −2.06 to −1.33). CPC was the most effective chemical intervention against *Campylobacter* as a dip treatment (*P* < 0.05). Hydrogen Peroxide (HP) was also effective as a post-chill immersion intervention. Only one study was included in this analysis. FA, PAA, SSS, and TSP also represent effective interventions at reducing *Campylobacter* concentrations. Each are statistically less effective than CPC (*P* < 0.05). Nevertheless, they are effective when compared to spray treatments. Several studies were included for PAA analysis representing moderate heterogeneity (I^2^ = 42 %, *P* > 0.05). ASC is the least effective intervention of immersion treatments.

**Fig. 10:**
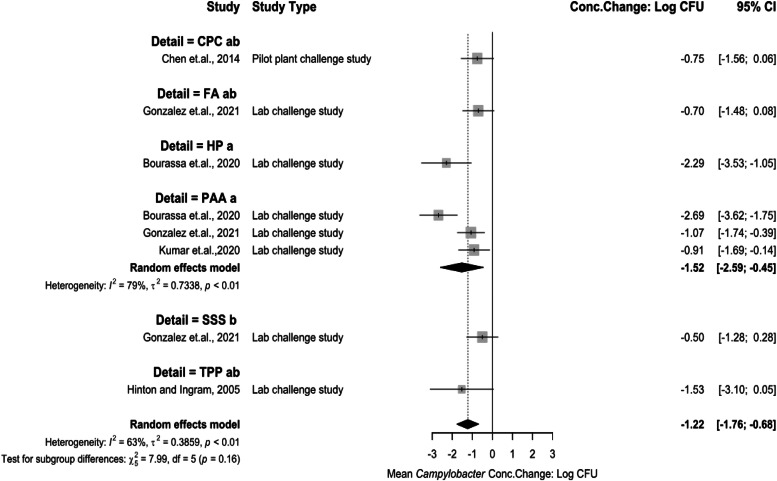
Campylobacter concentration change for spray interventions at post-chill stage. The random effects model results represent the mean concentration change of included spray interventions at the post-chill stage. Results > 0 indicate an increase in concentration. Results < 0 indicate a decrease. Results = 0 indicate no change. Distinct letters next to the treatment description indicate statistically significant differences (*P* < 0.05). Abbreviations: CPC = cetylpiridium chloride, FA = formic Acid, HP = hydrogen peroxide, PAA = peroxyacetic acid, SSS= sulfuric acid + sodium sulfate solution, TPP = tripotassium phosphate.

There were several chemical interventions analyzed for *Campylobacter* concentration change as post-chill spray treatments (Fig. 10). CPC being −0.75 log_10_ CFU/mL (95 % CI: −1.56 to 0.06), FA being −0.70 log_10_ CFU/mL (95 % CI: −1.48 to 0.08), HP being −2.29 log_10_ CFU/mL (95 % CI: −3.53 to −1.05), PAA being −1.52 log_10_ CFU/mL (95 % CI: −2.59 to −0.45), SSS being −0.50 log_10_ CFU/mL (95 % CI: −1.28 to 0.28), and TPP being −1.53 log_10_ CFU/mL (95 % CI: −3.10 to 0.05). All treatments were less effective as a spray treatment when compared to immersion treatments. SSS was statistically the least effective treatment (*P* < 0.05). PAA analysis representing high heterogeneity (I^2^ = 79 %, *P* > 0.05) indicating that the intervention application results should be interpreted carefully. Some subgroups in this meta-analysis included only a single study due to limited available literature, which reduces statistical power and increases the potential for bias, limiting the reliability and interpretability of the results. However, these results were still presented in the forest plot as a reference for the reader’s interest, highlighting potential research gaps in *Campylobacter* concentration changes during poultry processing.

### Exposure assessment

*Baseline Model and Model Validation.* The baseline model is defined as a basic commercial chicken processing plant in the U.S. including scalding, feather picking/rehang, evisceration, carcass washing by inside-outside bird washers (IOBW), immersion chilling, parts cut-up and grinding without reported interventions or chlorine use from the SR-MA. Grinding is not available in most chicken processing plants, but ground product is available in several further processed products (NCC, 2023). Ground products, such as mechanically separated chicken (MSC), often go to further processes that include a lethality step. Therefore, ground products are seldom included in raw ready-to-cook pathogen analysis. The input parameters for the baseline processing model are described in (Table 2).

**Table 2:** Input parameters for baseline model simulation from the SR-MA.

Processing Stage	Concentration Change Distribution	Unit
Receiving (Initial Concentration)	Normal(4.81,0.46)	log_10_ CFU/mL
Scalding	Normal(−2.86,0.66)	log_10_ CFU/mL
Feather Picking	Normal(1.17,0.45)	log_10_ CFU/mL
Evisceration	Normal(0.13,0.44)	log_10_ CFU/mL
Carcass Wash	Normal(−0.39,0.33)	log_10_ CFU/mL
Carcass Chill	Normal(−1.48,0.23)	log_10_ CFU/mL
Cut Up Parts	Normal(−0.58,0.16)	log_10_ CFU/mL
Comminuted Chicken	Normal(−0.35,0.26)	log_10_ CFU/mL

**Fig. 11:**
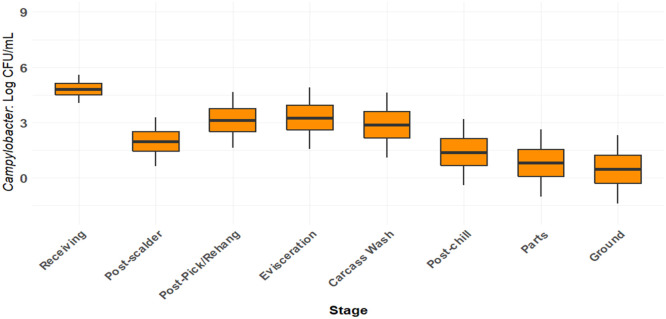
Baseline Campylobacter BIO-map. The chart represents Campylobacter concentrations per stage without interventions or chlorine.

The simulation estimated *Campylobacter* concentration to be incoming at 4.81 log_10_ CFU/mL (95 % CI: 4.05 to 5.57), at Scalding 1.95 log_10_ CFU/mL (95 % CI: 0.62 to 3.26), at Feather Picking 3.11 log_10_ CFU/mL (95 % CI: 1.62 to 4.63), at Evisceration 3.25 log_10_ CFU/mL (95 % CI: 1.57 to 4.91), at Carcass Wash 2.86 log_10_ CFU/mL (95 % CI: 1.09 to 4.62), and Whole Birds after Immersion Chill 1.38 log_10_ CFU/mL (95 % CI: −0.42 to 3.17). *Campylobacter* concentration after cut-up was estimated at 0.80 log_10_ CFU/mL (95 %CI: −1.02 to 2.61), and 0.45 log_10_ CFU/mL (95 % CI: −1.42 to 2.31) (Fig. 11). The model output suggests that a processing plant can reduce *Campylobacter* concentration up to 3 logs and be effective food safety control with minimal interventions. A rehang estimate could not be included. LC for rehang could not be calculated from the SR-MA due to limitations in the number of studies. Post-feather pick carcasses pass through steps, such as feet and hock cutters, followed by rehang. The time between stages last a few seconds so there is the possibility that *Campylobacter* concentration differences are minimal between stages, unless interventions are placed between stages. Therefore, feather picking and rehang steps are represented as one stage. Subsequent processing steps represent additional *Campylobacter* concentration reduction and mitigation of previous increases.

**Fig. 12:**
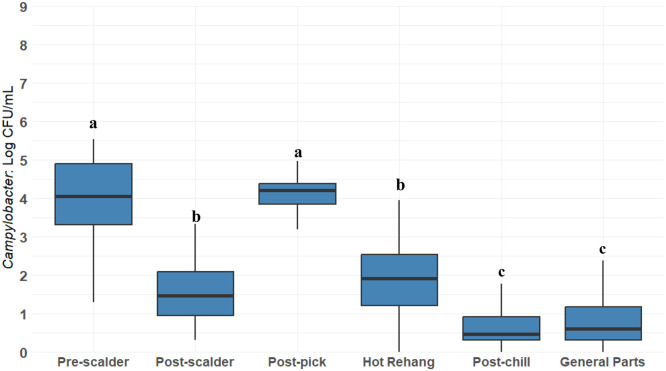
Commercial processing plant Campylobacter bio-map. This figure represents the concentrations obtained from commercial processing plants. The difference between concentrations between feather picking and rehang is because the processing plants include an intervention between stages. The type of intervention is undisclosed. The data provided by the commercial integrator was used for model validation. Distinct letters on top of the stage indicate statistically significant differences (*P* < 0.05).

Validation of the final *Campylobacter* concentration in chicken was done by comparing the model with the *Campylobacter* concentration estimates obtained from data collected from commercial processing plants in the U.S. (Fig. 12). *Campylobacter* concentrations recovered from pre-scalder (incoming concentration), post-scalder, post-pick, hot rehang, post-chill, and cut-up parts sampling were collected from routine testing of 31 commercial processing facilities in the U.S. over the period from 2018 to 2024. *Campylobacter* concentration was measured using various plating methods with a limit of detection (LOD) of 1 CFU/mL. The processing plants reported using PAA for chill, post-chill and cut-up processes.

The incoming concentration at the receiving stage was 4.04 log_10_ CFU/mL. The concentration after scalding fell to 1.46 log_10_ CFU/mL followed by an increase to 4.20 log_10_ CFU/mL after feather picking. The decrease after scalding was significantly different from receiving and the subsequent increase was significant when compared to the scalding stage (*P* < 0.05). The subsequent decrease to 1.90 log_10_ CFU/mL at the rehang stage was significantly different from the feather picking stage (*P* < 0.05). Most of the processing plants represented in the data include a PAA or Chlorine spray treatment prior to the rehang stage, significantly reducing *Campylobacter* concentrations (*P* < 0.05). Significant reductions were represented after post-chill where whole bird samples averaged *Campylobacter* concentrations of 0.45 log_10_ CFU/mL (*P* < 0.05). *Campylobacter* estimates for cut-up parts from the commercial processing data are 0.60 log_10_ CFU/mL. Subsequent steps such as cut up and deboning do not significantly change *Campylobacter* concentrations (*P* > 0.05). Data from comminuted products were not obtained due to product not tested because the company produces comminuted product for further processing, thus not subject to testing under USDA-FSIS regulation. Validation of *Campylobacter* concentrations for the baseline simulation of this product type could not be achieved.

**Fig. 13:**
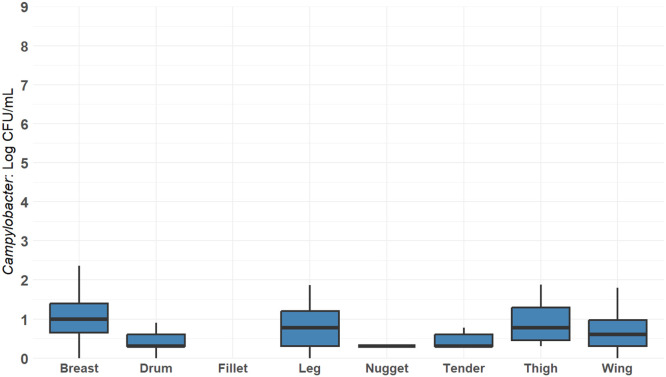
Commercial integrator *Campylobacter* concentrations per parts. This figure represents the general cut-up parts broken down by the categories obtained from the testing results obtained from commercial processing plants. All cut-up parts categories were treated with PAA.

Cut up parts data obtained from the commercial processing plants were organized in separate categories (Fig. 13). Results were pooled per category to compare *Campylobacter* concentration between parts. The concentration between parts resulted in boneless skinless breast 0.99 log_10_ CFU/mL, drumsticks 0.43 log_10_ CFU/mL, boneless fillets 0 log_10_ CFU/mL, legs 0.83 log_10_ CFU/mL, breast trim used for nuggets 0.30 log_10_ CFU/mL, tenders 0.67 log_10_ CFU/mL, bone-in thighs 0.94 log_10_ CFU/mL, and wings 0.70 log_10_ CFU/mL were compared for differences between cut-up parts. *Campylobacter* concentration estimates for parts were at or below 1 log_10_ CFU/mL. The concentration comparison between part types was not significant (*P* > 0.05). All parts were sampled after all PAA applications.

The *Campylobacter* concentration pattern observed in commercial plants (Fig. 12) was consistent with the baseline simulation model (Fig. 11) and SR-MA findings (Fig. 3), though absolute levels were lower due to prevalent PAA use.

*Scenario Analysis.* 3 different single intervention application methods (air chill, spray, and immersion) and 5 of the most studied chemical interventions (ASC, CPC, PAA, TPP, and TSP) were selected from the SR-MA to analyze its capacity of changing a processing plant’s ability to control *Campylobacter* in whole birds, cut-up parts or comminuted chicken for a total of 24 single intervention scenarios. The input parameters for each intervention scenario simulation are described in Table 3. Table 4 includes the *Campylobacter* concentrations and efficacy estimates for all scenarios for whole birds, cut-up parts and comminuted product respectively. *Campylobacter* concentrations were converted to CFU/mL because final concentration estimates were very low to accurately determine intervention efficacy. The baseline *Campylobacter* estimates for whole birds was 23.44 CFU/mL (95 % CI: 0.06 to 10715.19), 6.31 CFU/mL (95 % CI: 0.01 to 3801.89) for cut up parts, and 2.82 CFU/mL (95 % CI: 0 to 2290.87) for comminuted chicken.

**Table 3:** Input distributions of processing interventions from the SR-MA used for intervention efficacy analysis.

Intervention Type	Concentration Change Distribution	Unit
Pre-Chill Immersion	Normal(−1.11,0.45)	log_10_ CFU/mL
a. PAA	Normal(−1.13,0.47)	log_10_ CFU/mL
Pre-Chill Spray	Normal(−0.77,0.27)	log_10_ CFU/mL
a. PAA	Normal(−0.50,0.29)	log_10_ CFU/mL
b. CPC	Normal(−1.56,2.11)	log_10_ CFU/mL
Air Chiller	Normal(−1.05,0.54)	log_10_ CFU/mL
Post-Chill Immersion - Whole Birds	Normal(−1.87,0.21)	log_10_ CFU/mL
a. PAA	Normal(−1.79,0.25)	log_10_ CFU/mL
b. ASC	Normal(−0.68,0.57)	log_10_ CFU/mL
c. CPC	Normal(−4.29,0.85)	log_10_ CFU/mL
d. TSP	Normal(−1.69,0.46)	log_10_ CFU/mL
Post-Chill Spray - Whole Birds	Normal(−1.22,0.27)	log_10_ CFU/mL
a. PAA	Normal(−1.52,0.55)	log_10_ CFU/mL
b. CPC	Normal(−0.75,0.95)	log_10_ CFU/mL
c. TPP	Normal(−1.53,1.18)	log_10_ CFU/mL
Post-Cut Up Immersion	Normal(−1.87,0.21)	log_10_ CFU/mL
a. PAA	Normal(−1.79,0.25)	log_10_ CFU/mL
b. ASC	Normal(−0.68,0.57)	log_10_ CFU/mL
c. CPC	Normal(−4.29,0.85)	log_10_ CFU/mL
d. TSP	Normal(−1.69,0.46)	log_10_ CFU/mL
Post-Cut Up Spray	Normal(−1.22,0.27)	log_10_ CFU/mL
a. PAA	Normal(−1.52,0.55)	log_10_ CFU/mL
b. CPC	Normal(−0.75,0.95)	log_10_ CFU/mL
c. TPP	Normal(−1.53,1.18)	log_10_ CFU/mL

**Table 4:** Intervention efficacy analysis for single intervention scenarios.

Scenario	Whole Bird Concentration (CFU/mL)	Intervention Efficacy (%)	Parts Concentration (CFU/mL)	Intervention Efficacy (%)	Comminuted Concentration (CFU/mL)	Intervention Efficacy (%)
Baseline	23.44	-	6.31	-	2.82	-
Pre-Chill Immersion	1.95	91.68	0.50	92.06	0.23	91.87
a. PAA	1.78	92.41	0.47	92.59	0.21	92.59
Pre-Chill Spray	4.07	82.62	1.07	83.02	0.48	83.02
a. PAA	7.59	67.64	2.00	68.38	0.89	68.38
b. CPC	0.07	99.72	0.17	97.25	0.08	97.25
Air Chiller	64.57	Not effective	16.98	Not effective	7.59	Not effective
Post Chill Immersion Whole Birds	0.35	98.52	0.09	98.59	0.04	98.55
a. PAA	0.42	98.22	0.11	98.30	0.05	98.26
b. ASC	5.25	77.61	1.32	79.11	0.60	78.62
c. CPC	0.00	99.99	0.00	99.99	0.00	99.99
d. TSP	0.51	97.81	0.13	97.91	0.06	97.86
Post Chill Spray Whole Birds	1.55	93.39	0.39	93.83	0.18	93.69
a. PAA	0.78	96.69	0.19	96.91	0.09	96.84
b. CPC	4.57	80.50	1.15	81.80	0.52	81.38
c. TPP	0.76	96.76	0.19	96.91	0.09	96.91
Post-Cut Up Immersion	-	-	0.35	94.50	0.16	94.38
a. PAA	-	-	0.42	93.39	0.19	93.24
b. ASC	-	-	5.25	16.82	2.34	16.82
c. CPC	-	-	0.00	99.98	0.00	99.98
d. TSP	-	-	0.51	91.87	0.23	91.68
Post-Cut Up Spray	-	-	1.55	75.45	0.69	75.45
a. PAA	-	-	0.78	87.70	0.35	87.70
b. CPC	-	-	4.57	27.56	2.04	27.56
c. TPP	-	-	0.76	87.98	0.35	87.70

Pre-chill and Post-chill applications were added to the baseline simulation as single intervention. PAA as pre-chill immersion had an efficacy of 92.41 % reduction for whole birds, 92.59 % for cut-up parts, and comminuted chicken. PAA as a pre-chill spray had an efficacy of 67.64 % for whole birds, 68.38 % for cut-up parts, and comminuted chicken. CPC as a pre-chill spray has an efficacy of 99.72 % for whole birds, 97.25 % in cut-up parts, and comminuted chicken.

Air chilling was included as an alternative chilling stage. Air chill is predominantly used in Europe, but it has been implemented in various U.S. processing plants. Air chilling was not effective at reducing *Campylobacter* concentration when compared to the baseline model utilizing immersion chilling. The *Campylobacter* concentration estimates of 64.57 CFU/mL in whole birds, 16.98 CFU/mL in parts, and 7.59 CFU/mL in comminuted chicken represent a higher concentration when compared to the baseline estimates for each category.

Post-chill applications were effective at reducing *Campylobacter* concentration. Post-chill dip treatment had an efficacy of 98.52 % in whole birds, 98.59 % in cut-up parts, and 98.55 % for comminuted chicken. Efficacies of 94.50 % for cut-up parts, and 94.38 % for comminuted chicken respectively were achieved by applying an intervention post-cut-up. Post-chill spray application on whole bird carcasses had efficacies of 93.39 % in whole birds, 93.83 % on cut-up parts, and 93.69 % on comminuted chicken. Efficacies of 75.45 % were achieved for cut-up parts and comminuted chicken if the spray interventions were applied at post-cut-up.

All antimicrobial chemicals were effective as a post-chill treatment. CPC as a post-chill immersion treatment had an efficacy of 99.99 % in whole birds, cut-up parts, and comminuted chicken when it’s applied to whole bird carcasses post- chill. CPC as an immersion treatment had an efficacy of 99.98 % when applied post-cut-up. However, CPC as a post-chill spray had an efficacy of 80.50 % in whole birds, 81.80 % in cut-up parts, and 81.38 % in comminuted chicken. CPC as a spray application had an efficacy of 27.56 % for cut-up parts and comminuted chicken when applied post-cut-up.

PAA as a post-chill immersion application had an efficacy of 98.22 % in whole birds, 98.30 % in cut-up parts, and 98.26 % in comminuted chicken. PAA as an immersion application post-cut-up intervention had an efficacy of 93.39 % in cut-up parts, and 93.24 % in comminuted chicken. PAA as a post-chill spray application had an efficacy of 96.69 % in whole birds, 96.91 % for cut-up parts, and 96.84 % for comminuted chicken. PAA as a spray application post-cut-up had an efficacy of 87.70 % for cut-up parts and comminuted chicken. TSP and TPP are also effective antimicrobials as immersion and spray treatment. ASC is the least effective overall as a post-chill immersion treatment.

Intervention efficacy analyses were performed by incorporating PAA spray and dip applications at multiple processing stages. Results suggest that *Campylobacter* concentrations can be at or near undetectable levels when a combination of interventions are applied in the process (Data not shown).

## Discussion

### Meta-analysis for baseline campylobacter concentration per processing stage

The SR-MA identified the stage-specific variation in *Campylobacter* concentration across processing. Immersion chilling significantly reduces *Campylobacter* concentrations on whole bird carcasses and cut-up parts. The SR-MA demonstrates that the chilling and subsequent stages have the capability of reducing *Campylobacter* concentrations with minimal to no intervention, aligned with global bio-mapping studies from commercial processing plants (Betancourt-Barszcz et al., 2024; Chavez-Velado et al., 2024; DeVillena et al., 2022; Kingsbury et al., 2023; Vargas et al., 2023). However, establishing baseline *Campylobacter* concentration for comminuted products directly from the SR-MA was precluded by a lack of before-after studies from commercial processing facilities. Baseline simulation using LC levels estimated *Campylobacter* concentration for comminuted chicken, suggesting reductions achieved during immersion chilling and the cutting-up stage can be sustained through a grinding process if growth preventing conditions (e.g. time and temperature) are maintained. This estimate remains unvalidated against commercial processing or with published studies, primarily because most U.S. ground poultry undergoes further processing (e.g., thermal inactivation) and is exempt from the regulatory sampling as it’s not sold raw.

The SR-MA to establish baseline values presented limitations. The heterogeneity and within study variation was low for both concentration and concentration change analysis. The study required dividing the studies into several subgroups and even though a mean value was established and was verified by comparisons to commercial sampling, the subgroup analysis had very few studies with sufficient data points to perform within study and between study variation analysis.

### Meta-analysis for interventions against Campylobacter

Most analyzed interventions could reduce *Campylobacter* concentration on carcasses and on cut-up parts, but evidence is often limited to single studies, precluding robust assessment of heterogeneity. For example, only two studies evaluated cloacal plugs, which limited cross-contamination during feather picking compared to controls; more research is needed to optimize such novel approaches(Berrang et al., 2018, 2001).

Heterogeneity was higher for post-chill chemical interventions, reflecting greater study number but varied methodologies (sampling, matrices). PAA emerged as the dominant U.S. intervention, applied via immersion or spray pre- or post- carcass chiller. PAA is also widely used in carcass chillers, but none of the studies obtained from the SR-MA observed dwell times like a typical immersion chiller in processing plants and it was decided to limit PAA application studies to pre-chill or post-chill. The results for PAA are consistent to past reviews (Cano et al., 2021; Oyarzabal, 2005). The decision to use PAA over other available chemical antimicrobials is due to cost-effectiveness and implementability.

Lesser-known interventions, such as cloacal plugs, cloacal washes, UV, etc. were included to for comparison, they require further research for optimization and potential integration as complementary controls.

### Exposure assessment

The baseline simulation model captured *Campylobacter* concentration patterns observed in the SR-MA and the validation data, though simulated concentrations for whole birds and parts were higher than commercial validation data. This discrepancy is likely attributable to widespread PAA use in commercial processing plants, absent in the baseline model. Importantly, incorporating interventions into the model yielded concentrations matching validation data, confirming the baseline as a valid representation of a U.S. plant without interventions. This validated model provides a foundation for simulating diverse intervention scenarios.

The key model finding indicate low post-chill *Campylobacter* concentrations are maintained through the cut-up and grinding process. Further reductions for cut-up parts and comminuted chicken are attributed to temperature control (inhibiting growth) and sanitation (minimizing cross-contamination). While interventions like PAA significantly reduce *Campylobacter* concentrations pre-cut, they show limited additional reduction post-cut. Applying interventions at the cut-up stage helps maintain low levels and may confer carryover effects to subsequent stages like grinding or packaging. Notably, post-processing concentrations were similar across cut-up parts type and comparable to whole carcasses, suggesting uniform exposure risk regardless of part. Simulated comminuted product concentrations serve as a crucial benchmark due to the lack of comparable commercial data.

Simulating a chicken processing plant presented several limitations. Model development faced data scarcity, particularly for cut parts and comminuted products, due to limited commercial/ pilot plant trials and restricted access for controlled studies (e.g., testing reduced/ no interventions was precluded by regulations). Some uncertainties include the sampling locations and sampling matrices to determine initial concentration. Even though the initial concentration was comparable to initial *Campylobacter* concentrations obtained from commercial processing plants, the SR-MA contained studies where incoming load was determined by sampling at different locations before the scalder and sampled several matrices from carcass rinses to cecal content (Berghaus et al., 2013; Berrang, Buhr, et al., 2000; Kotula and Pandya, 1995; Mead et al., 1995; Potturi-Venkata et al., 2007; Stern and Robach, 2003). Another uncertainty is the impact of cross contamination on *Campylobacter* levels throughout the process. Cross contamination in feather picking is known to occur, but the rate of cross-contamination per bird and how much equipment contributes to cross contamination throughout subsequent steps was not factored in the simulation process perhaps underestimating *Campylobacter* concentrations in the model. The effect on the different sampling locations could not be included. A source of variability included the wide range of sample rinse volumes, detection methods, and sample matrices. Carcass rinse data extracted from the studies ranged from 200 mL to 400 mL. There are differences in the amount of *Campylobacter* concentrations obtained from different rinse volumes (Williams et al., 2010). The type of rinse and the type of enrichment and plating method have different sensitivities that may influence concentration data from studies (Gonsalves et al., 2016; Hiett, 2017; Line et al., 2001). The SR-MA was sufficient in providing data for the simulation models.

### Intervention efficacy analysis

The intervention efficacy analysis confirmed that single interventions can significantly reduce *Campylobacter* concentrations. Air chilling was included in the scenario analysis as an alternative chilling stage. Air chilling was ineffective for *Campylobacter* control, necessitating validation of complementary pre-/ post-chill interventions. Post-chill immersion interventions, particularly PAA and CPC, were the most effective interventions. Pre-chill interventions are less effective than post-chill interventions. *Campylobacter* is present at higher levels and chicken carcasses may not be exposed to an intervention for long periods of time prior to chilling. Intestinal content, debris, or tissues that contain high levels of *Campylobacter* may still be present at pre-chill stages. Post-chill interventions are applied after all mayor *Campylobacter* contamination sources like feathers, viscera, and intestinal content have been removed and the carcasses have gone through a washing process to remove all visible debris. This allows the chemical to act on the actual product and not compete with other organic material that can lower its effectiveness. There may be a carryover factor in these results that may overestimate the results. Chemical interventions, like PAA, are the last hurdle that is applied in a commercial processing setting before packaging or grinding.

The multiple intervention scenarios analyzing the application of PAA resulted in undetectable concentrations of *Campylobacter*. However, these levels are overestimated, and these concentration levels may not reflect a real-world scenario. It is important to note that single interventions are effective, but a multi-hurdle system, including pre-chill and post-chill interventions is most effective against *Campylobacter*.

The single intervention and multiple interventions model present *Campylobacter* concentrations after chemical interventions comparable to the commercial processing bio-map. These results validate that the baseline model without interventions presents a possible outcome of *Campylobacter* concentrations if interventions are not applied and it can be used as a starting point to model future exposure assessments modules. However, the model presents several limitations. Much of the data utilized for intervention efficacy analysis was extracted from a limited number of studies. PAA is the most used chemical antimicrobial in processing thus more studies are available, particularly for pre- and post-chill applications. Models studying the variations in immersion chilling will allow for better assessments. The limited number of studies limits the amount of data points used to reduce variation in the results. The general risk assessment model only analyzed overall intervention data. It did not consider different concentrations of the chemical, contact time and pH levels, which can influence the effectiveness of many of these interventions. Other lesser-known interventions from the SR-MA were not included because of the limited number of studies available, and commonly used chemicals were only considered for this analysis.

In conclusion, the SR-MA enabled construction of a validated baseline model simulating *Campylobacter* concentrations in U.S. processing plants *without* interventions. Key implications:

• Process Control: Significant reductions occur naturally during immersion chilling and are maintained through cutting/grinding via temperature control and sanitation.
• Intervention Strategy: Post-chill chemical applications (PAA/CPC) are optimal. Multi-hurdle approaches minimize cross-contamination and reduce part-to-part variability, yielding uniform concentrations (often <1 log₁₀ CFU/mL) across all products.
• Uniform Exposure Risk: Finished products (whole carcasses, parts, comminuted) pose comparable *Campylobacter* exposure risk due to concentration equalization during processing.
• Critical Data Gap: Cut parts dominate U.S. consumption yet remain underrepresented in risk assessments. Comminuted products lack commercial validation data.

While multi-hurdle interventions lower risk, they cannot eliminate *Campylobacter*. Future work must focus on a) Validating interventions for air-chilled products, b) Expanding cut-part and comminuted product sampling, c) Assessing novel chemical/non-chemical interventions and d) Evaluating pre- and post-processing mitigation steps.

## CRediT authorship contribution statement

**Rafael E. Rivera:** Conceptualization, Data curation, Formal analysis, Funding acquisition, Investigation, Methodology, Project administration, Resources, Software, Supervision, Validation, Visualization, Writing – original draft, Writing – review & editing. **Jinquan Wang:** Formal analysis, Methodology, Software, Validation, Visualization, Writing – original draft, Writing – review & editing. **Abhinav Mishra:** Formal analysis, Methodology, Software, Supervision, Validation, Visualization. **Harshavardhan Thippareddi:** Conceptualization, Formal analysis, Project administration, Resources, Supervision, Writing – original draft, Writing – review & editing. **Sanjay Kumar:** Writing – review & editing. **Manpreet Singh:** Resources, Supervision, Writing – original draft.

## Disclosures

The authors declare the following financial interests/personal relationships which may be considered as potential competing interests: Rafael E Rivera Betancourt reports administrative support was provided by US Poultry and Egg Association. If there are other authors, they declare that they have no known competing financial interests or personal relationships that could have appeared to influence the work reported in this paper.
